# Homeopathy effects in patients during oncological treatment: a systematic review

**DOI:** 10.1007/s00432-022-04054-6

**Published:** 2022-06-22

**Authors:** Anna Wagenknecht, Jennifer Dörfler, Maren Freuding, Lena Josfeld, Jutta Huebner

**Affiliations:** Klinik Für Innere Medizin II, Hämatologie und Internistische Onkologie, Universitätsklinikum Jena, Am Klinikum 1, 07747 Jena, Germany

**Keywords:** Homeopathy, Cancer, Complementary alternative medicine, Oncological treatment, Adverse events

## Abstract

**Purpose:**

In this systematic review we included clinical studies from 1800 until 2020 to evaluate evidence of the effectiveness of homeopathy on physical and mental conditions in patients during oncological treatment.

**Method:**

In February 2021 a systematic search was conducted searching five electronic databases (Embase, Cochrane, PsychInfo, CINAHL and Medline) to find studies concerning use, effectiveness and potential harm of homeopathy in cancer patients.

**Results:**

From all 1352 search results, 18 studies with 2016 patients were included in this SR. The patients treated with homeopathy were mainly diagnosed with breast cancer. The therapy concepts include single and combination homeopathic remedies (used systemically or as mouth rinses) of various dilutions. Outcomes assessed were the influence on toxicity of cancer treatment (mostly hot flashes and menopausal symptoms), time to drain removal in breast cancer patients after mastectomy, survival, quality of life, global health and subjective well-being, anxiety and depression as well as safety and tolerance. The included studies reported heterogeneous results: some studies described significant differences in quality of life or toxicity of cancer treatment favouring homeopathy, whereas others did not find an effect or reported significant differences to the disadvantage of homeopathy or side effects caused by homeopathy. The majority of the studies have a low methodological quality.

**Conclusions:**

For homeopathy, there is neither a scientifically based hypothesis of its mode of action nor conclusive evidence from clinical studies in cancer care.

**Supplementary Information:**

The online version contains supplementary material available at 10.1007/s00432-022-04054-6.

## Introduction

Cancer embodies one of the leading causes of death; morbidity and mortality due to cancer are increasing steadily (Radtke 2022). Receiving the diagnosis, many patients are desperate and try additional treatment to their standard cancer therapy. More than 25% of the general population in Europe is using complementary and alternative medicine (CAM) regularly on less severe health conditions such as neck pain or allergies (Laura et al. 2018), prescribed by some physicians as a placebo with few side effects. Faced with a cancer diagnosis, many patients revert to the use of CAM. Homeopathy is a CAM system that, globally, became more and more popular over the past decades. Based on the “Law of Similars” by the German physician Samuel Hahnemann, homeopaths assume that a substance, which causes certain effects, can also be used to treat them if prescribed in a very low dosage (Shah 2018). Therefore, homeopathic remedies (e.g. plant, animal or mineral) are diluted to so-called potencies. In classical homeopathy, these steps of dilution (1:10; 1:100 or 1:50.000) are repeated so many times that there is not a single molecule of the substance left in the remedy (Tschech 2022). Nevertheless, homeopaths are convinced of the effectiveness of homeopathic treatments, while science expresses criticism and doubt. Some explanation attempts for the mode of action of homeopathy are nanoparticles and water memory, but none of these were verified through clinical studies yet (Fritzsche 2011; Nuhn 2005).

The most common dilution (1:100) is the C- potency, or, using Hahnemann’s dilution method, CH-potency. Repeating this dilution-method for a second time creates a C2-potency (1:10.000). C2 diluted again results in a C3-potency and so forth (Genneper 2017). There exist three main approaches to homeopathic prescribing: in the individualised or classical homeopathy single remedies are used depending on the patients individual condition and history, in the clinical homeopathy the same remedy is used for a group of patients with specific conditions and in the complex homeopathy a number of remedies is used in a defined combination for particular symptoms (Pérol et al. 2012).

Due to the controversial discussions on homeopathic therapies, a wide variety of publications exists addressing this matter. But for homeopathy being such a popular treatment method, there are surprisingly few clinical studies, systematic reviews (SRs) or meta-analyses, and only of limited quality. Likewise, only a few studies examine the influence of homeopathy on carcinoma, while homeopathy is frequently used against the toxicity of cancer treatments and even for its cure. Therefore, SRs and an extensive evaluation of clinical studies are needed to provide high-level evidence of the effects of homeopathy in cancer patients.

## Methods

### Criteria for including and excluding studies in the review

Inclusion and exclusion criteria are listed in Table 1 based on a PICO- model. Generally, all study types were included if they reported patient-relevant outcomes after guideline-based treatment of adult cancer patients with any intervention containing homeopathy. Because of the wide range of application fields, all cancer entities were included. Since little high-quality evidence was expected, systematic reviews and randomized controlled trials were included as well as controlled trials, one-armed studies and retrospective studies. Criteria for rejecting studies were primary prevention, grey literature, other publication type than primary investigation/report (e.g. comments, letters, abstracts) and study population with children (under the age of 18) or precancerous conditions, if results or numeral details of adult patients with cancer were not reported separately. Additionally, studies were excluded if they reported no patient centred outcomes (laboratory parameters except Prostate Specific Antigen (PSA) which is a valuable parameter for cancer progression of prostate cancer). Language restrictions were made to English and German.

**Table 1:** Inclusion and exclusion criteria

PICO	Inclusion criteria	Exclusion criteria
Patients	Cancer patients (all entities and stages) Adult patients (age > 18) Guideline-based cancer treatment	Preclinical studies Study population with children or only precancerous conditions, if numeral details are not reported separately Primary prevention
Intervention	Every intervention containing homeopathy No restrictions regarding the type of homeopathy, dose, mode of application	
Comparison	All possible control groups (active control, placebo, standard care, observation)	
Outcome	Influence on toxicity of cancer treatment (skin reaction, nausea and vomiting, joint pain and stiffness, oral mucositis) Time to drain removal Survival Menopausal symptoms Quality of life and other psychological outcomes Sleep Safety and side effects	No patient centred outcomes (e.g. laboratory parameters)
Others	All study types (including SRs, RCTs, CTs, one-armed studies and retrospective studies) Language: German and English Full publication	Other publication type than primary investigation/report Case reports Gray literature (conference articles, abstracts, comments, letters, ongoing studies, unpublished literature, etc.) Full text not available in German or English

### Study selection

A systematic research was conducted using five databases (Medline (Ovid), CINAHL (EBSCO), EMBASE (Ovid), Cochrane CENTRAL and PsycINFO (EBSCO)) in February 2021. For each of these databases a complex search strategy was developed consisting of a combination of MeshTerms, keywords and text words in different spellings connected to cancer and homeopathic therapy (Table 2). The search string was highly sensitive, since it was not restricted by filters of study or publication type. After importing the search results into EndNote X9, all duplicates were removed and a title- abstract- screening was carried out by two independent reviewers (AW and JD). In case of disagreement consensus was made by discussion or a third reviewer was consulted (JH). After that, all full texts were retrieved and screened again independently by both reviewers. When title and abstract did not have sufficient information for screening purposes, a full-text copy was retrieved as well. Additionally bibliography lists of all retrieved articles were searched for relevant studies.

**Table 2:** Search Strategy

Datenbase	Search Strategy
Ovid Medline	1. Homeopathy/or homeopath$.mp. or homoepath$.mp 2. exp neoplasms/or neoplasm$.mp or cancer$.mp. or tumo?r$.mp. or malignan$.mp. or oncolog$.mp. or carcinom$.mp. or leuk?emia.mp. or lymphom$.mp. or sarcom$.mp 3. 1 AND 2 4. Limit 3 to English or limit 3 to German 5. Limit 4 to yr = "1800 -1995″ **OR** limit 4 to yr = ”2018-current” 6. (5 and humans/) or (5 not animals/) 7. ((((comprehensive* or integrative or systematic*) adj3 (bibliographic* or review* or literature)) or (meta-analy* or metaanaly* or "research synthesis" or ((information or data) adj3 synthesis) or (data adj2 extract*))).ti,ab. or (cinahl or (cochrane adj3 trial*) or embase or medline or psyclit or (psycinfo not “psycinfo database”) or pubmed or scopus or “sociological abstracts” or “web of science” or central).ab. or ("cochrane database of systematic reviews" or evidence report technology assessment or evidence report technology assessment summary).jn. or Evidence Report: Technology Assessment*.jn. or (network adj1 analy*).ti,ab.) or (((review adj5 (rationale or evidence)).ti,ab. and review.pt.) or meta-analysis as topic/ or Meta-Analysis.pt.) 8. Randomi?**ed controlled trial?.pt. or controlled clinical trial?.pt. or randomi?***ed.ti,ab.or placebo.ti,ab. or drug therapy.sh. or randomly.ti,ab. or trial?.ti,ab. or group?.ti,ab 9. 6 AND (7 OR 8) 10. 6 NOT 9
Ovid Embase	1. Homeopathy/ or homeopath$.mp. or homoepath$.mp 2. Exp neoplasm/ or neoplasm$.mp or cancer$.mp. or tumo?r$.mp. or malignan$.mp. or oncolog$.mp. or carcinom$.mp. or leuk?emia.mp. or lymphom$.mp. or sarcom$.mp 3. 1 AND 2 4. Limit 3 to English or limit 3 to German 5. Limit 4 to yr = ”1800–1995” **OR** limit 4 to yr = ”2018-current” 6. (5 and humans/) or (5 not animals/) 7. ((((Comprehensive* or integrative or systematic*) adj3 (bibliographic* or review* or literature)) or (meta-analy* or metaanaly* or “research synthesis” or ((information or data) adj3 synthesis) or (data adj2 extract*))).ti,ab. or (cinahl or (cochrane adj3 trial*) or embase or medline or psyclit or (psycinfo not “psycinfo database”) or pubmed or scopus or “sociological abstracts” or “web of science” or central).ab. or (“cochrane database of systematic reviews” or evidence report technology assessment or evidence report technology assessment summary).jn. or Evidence Report: Technology assessment*.jn. or (network adj1 analy*).ti,ab.) or (exp Meta Analysis/or ((data extraction.ab. or selection criteria.ab.) and review.pt.)) 8. Crossover procedure/or double-blind procedure/or randomized controlled trial/or -lind procedure/or (random$ or factorial$ or crossover$ or (cross adj1 over$) or placebo$ or (doubl$ adj1 blind$) or (singl$ adj1 blind$) or assign$ or allocat$ or volunteer$).ti,ab,de 9. 6 AND (7 OR 8) 10. 6 NOT 9
Cochrane	**#**1. [mh homeopathy] or homeopath* or homoepath* **#**2. [mh neoplasms] or neoplasm* or cancer? or tum*r? or malignan* or oncolog* or carcinom* or leuk*mia or lymphoma? or sarcoma? **#**3. **#**1 AND #2
Ebsco—PsychINFO	S1. Homeopath* OR homoepath* S2. ((DE “Neoplasms” OR DE “Benign Neoplasms” OR DE “Breast Neoplasms” OR DE “Endocrine Neoplasms” OR DE “Leukemias” OR DE “Melanoma” OR DE “Metastasis” OR DE “Nervous System Neoplasms” OR DE “Terminal Cancer”) OR (TX neoplasm* OR TX cancer OR TX tumo#r OR TX malignan* OR DE „oncology “ OR TX oncolog* OR TX carcinom* OR TX leuk#emia OR TX lymphoma OR TX sarcoma)) S3. (LA German OR LA English) S4. S1 AND S2 AND S3 S5. ((Comprehensive* OR integrative OR systematic*) N3 (bibliographic* OR review* OR literature)) OR (meta-analy* or metaanaly* or “research synthesis” OR ((information OR data) N3 synthesis) OR (data N2 extract*)) OR ((review N5 (rationale OR evidence)) AND DE “Literature Review”) OR (AB(cinahl OR (cochrane N3 trial*) OR embase OR medline OR psyclit OR pubmed OR scopus OR “sociological abstracts” OR “web of science” OR central)) OR DE “Meta Analysis” OR (network N1 analy*) S6. DE “Treatment Effectiveness Evaluation” OR DE “Treatment Outcomes” OR DE "Psychotherapeutic Outcomes" OR DE "Placebo" or DE "Followup Studies" OR placebo* OR random* OR "comparative stud*" OR (clinical N3 trial*) OR (research N3 design) OR (evaluat* N3 stud*) OR (prospectiv* N3 stud*) OR ((singl* OR doubl* OR trebl* OR tripl*) N3 (blind* OR mask*) S7. S4 AND (S5 OR S6) S8. S4 NOT S7
Ebsco- CINAHL	S1. MH “Homeopathy” OR TX homeopath* OR TX homoepath* S2. (MH “Neoplasms + ” OR TX neoplasm* OR TX cancer OR TX tumo#r OR TX malignan* OR TX oncolog* OR TX carcinom* OR TX leuk#emia OR TX lymphoma OR TX sarcoma) S3. (LA German OR LA English) S4. S1 AND S2 AND S3 S5. (TI (systematic* n3 review*)) or (AB (systematic* n3 review*)) or (TI (systematic* n3 bibliographic*)) or (AB (systematic* n3 bibliographic*)) or (TI (systematic* n3 literature)) or (AB (systematic* n3 literature)) or (TI (comprehensive* n3 literature)) or (AB (comprehensive* n3 literature)) or (TI (comprehensive* n3 bibliographic*)) or (AB (comprehensive* n3 bibliographic*)) or (TI (integrative n3 review)) or (AB (integrative n3 review)) or (JN “Cochrane Database of Systematic Reviews”) or (TI (information n2 synthesis)) or (TI (data n2 synthesis)) or (AB (information n2 synthesis)) or (AB (data n2 synthesis)) or (TI (data n2 extract*)) or (AB (data n2 extract*)) or (TI (medline or pubmed or psyclit or cinahl or (psycinfo not “psycinfo database”) or “web of science” or scopus or embase)) or (AB (medline or pubmed or psyclit or cinahl or (psycinfo not “psycinfo database”) or “web of science” or scopus or embase or central)) or (MH “Systematic Review”) or (MH “Meta Analysis”) or (TI (meta-analy* or metaanaly*)) or (AB (meta-analy* or metaanaly*)) or network n1 analy* S6. (MH “Clinical Trials + ”) or PT Clinical trial or TX clinic* n1 trial* or TX ((singl* n1 blind*) or (singl* n1 mask*)) or TX ((doubl* n1 blind*) or (doubl* n1 mask*)) or TX ((tripl* n1 blind*) or (tripl* n1 mask*)) or TX ((trebl* n1 blind*) or (trebl* n1 mask*)) or TX randomi* control* trial* or (MH “Random Assignment”) or TX random* allocat* or TX placebo* or MH “Placebos”) or MH “Quantitative Studies”) or TX allocat* random* S7. S4 AND (S5 OR S6) S8. S4 NOT S7

### Assessment of risk of bias and methodological quality

All characteristics were assessed by two independent reviewers (AW and JD). In case of disagreement a third reviewer was consulted (JH) and consensus was made by discussion.

The risk of bias in the included studies was analysed with the SIGN- Checklist (“https://www.sign.ac.uk/what-we-do/methodology/checklists/”) for controlled trials Version 2.0 and IHE Quality Appraisal Checklist for Case Series Studies (“http://sandbox.ihe.ca/research-programs/methodology-development/case-series-studies-quality-appraisal/cssqac-about”). In addition, blinding of researchers, blinding of outcome assessment and comparability of groups before treatment, not only in terms of demographic variables but also concerning the outcomes, was examined.

The included studies were rated with the Oxford criteria. Additional criteria concerning methodology were size of population, application of power analysis, dealing with missing data and drop-out (report of drop-out reasons, application of intention-to-treat-analysis), adequacy of statistical tests (e.g. control of premises or multiple testing) and selective outcome reporting (report of all assessed outcomes with specification of statistical data as the p-value).

### Data extraction

Data extraction was performed by one reviewer (AW) and controlled by two independent reviewers (JD, JH). As a template for data extraction, the evidence tables from the National Guideline on Complementary and Alternative Medicine in Oncological Patients of the German Guideline Program in Oncology (“https://www.leitlinienprogramm-onkologie.de/english-language/”) were used. Concerning systematic reviews, only data from primary literature meeting the inclusion criteria of the present work were extracted.

## Results

The systematic search revealed 1352 results. No study was added by hand search. At first, duplicates were removed leaving 1007 studies. After screening title and abstract, 110 studies remained to complete review.

**Fig. 1:**
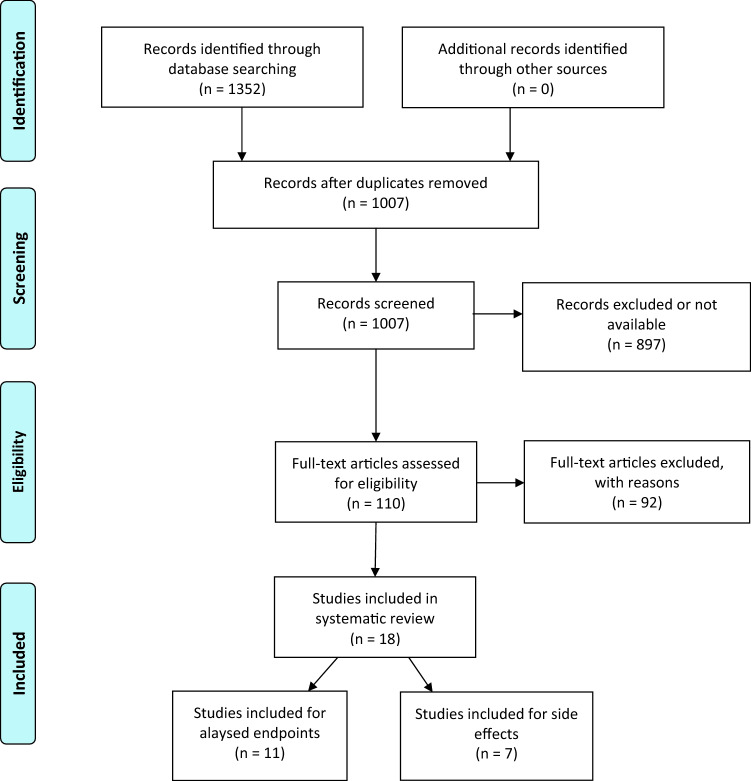
Flow Diagram

Finally, 18 publications were considered relevant due to the inclusion criteria of this present work and were included in this SR. We included 11 studies for endpoints: 0 SRs, 9 randomized controlled trials (RCTs) (Balzarini et al. 2000; Frass et al. 2015; Frass et al. 2020a, b; Heudel et al. 2019; Jacobs et al. 2005; Lotan et al. 2020; Luca Sorrentino 2017; Pérol et al. 2012; Thompson et al. 2005) and 2 controlled trials (CTs) (Karp et al. 2016; Steinmann et al. 2012) which investigated the efficacy of homeopathic treatment in cancer therapy. These studies were heterogeneous in terms of the assessed homeopathic intervention and cancer type. Additional seven studies were included only for safety and side effects due to severe lack of methodical and reporting quality (one uncontrolled three-armed pilot outcome study, five prospective single-armed studies and one single-armed retrospective study). The majority of studies observed breast cancer patients, the most common primary endpoint was influence of homeopathic treatment on toxicity of cancer treatment and one of the most frequent secondary endpoints was QoL. Detailed characterization of the included studies may be seen in Table 3. The flow of studies through the review can be seen in Fig. 1.

**Table 3:** Characterization of the included studies

Reference	Study type	CA type/Intervention/arms	Endpoints	Outcomes
Balzarini et al. (2000)	RCT	Breast-CA **arm A**: RTX + Belladonna 7CH globules + X-ray globules **arm B**: RTX + placebo	**1:** severity of skin reaction associated with RTX **2:** severity of skin reaction while RTX **3:** severity of skin reaction post RTX (15/30 d after) **AEs**	**1:** No significant differences (nsd) in skin colour or hyperpigmentation, significant differences regarding temperature to the touch in 4 of 8 defined times (A < B: T3: *p = *0.008, T4: *p = *0.016, T6: *p = *0.023, T7: *p = *0.011), significant differences regarding oedema (A > B: T5 and T6: *p = *0.025) **2:** nsd **3:** nsd **AEs:** 1 drop-out due to homeopathic exacerbation, 4 drop-outs due to the AE’s of radiation
Frass et al. 2015	RCT	Different types of CA, **arm A:** CTX or RTX + indiv. Homeopathy **arm B:** CTX or RTX	**1:** global health **2:** subjective well-being **3:** functioning scales **4:** side effcts of CTX/RTX (EORTC QLQ-C30: function-, sympt- sclaes) **AEs**	**1:** nsd for global health (95% KI = 2.3, 13.0; *p = *0.005) **2:** significant differences favouring arm A (95% KI = 8.5, 21.0; *p < *0.001) **3:** significant differences favo***ring arm A regarding physical functioning (95% KI = 8.6, 18.4; *p < *0.001), role functionig (95% KI = 0.4, 16.9; *p = *0.004), cognitive functioning (95% KI = 7.7, 19.7; *p < *0.001), social functioning (95% KI = 6.7, 20.4; *p < *0.001), emotional functioning (95% KI = 8.0, 20.7; *p < *0.001); significant differences favouring arm A regarding fatigue (95% KI = − 24.7, − 12.4; *p < *0.001), pain (95% KI = − 23.8, − 10.1; *p < *0.001), dyspnea (95% KI = − 19.6, − 4.3; *p = *0.002) and insomnia (95% KI = − 15.6, − 0.9; *p = *0.029) **4:** significant differences favouring arm A ragarding appetite loss (95% KI = − 17.1, − 2.7; *p = *0.007), nsd regarding nausea & vomiting, obstipation or diarrhoea **AEs:** no AEs of the homeopathic treatment observed
Jacobs et al. (2005)	RCT	Breast-CA with HF **arm A:** single indiv. Homeopathy + placebo combination **arm B:** single placebo + combination Homeopathy (Hyland’s Menopause) **arm C:** single placebo + combination placebo	**1:** HF severity score **2:** HF frquency (total number) **3:** Kupperman Menopausal Index **4:** QoL **AEs**	**1:** in a subgroup without tamoxifen significant differences to the disadvantage of arm B compared to arm A (95% KI -51.9, − 15.0; *p < *0.001) and arm C (95% KI 6.2, 47.1; *p = *0.01), no other significant differences in HF severity score **2:** in a subgroup without tamoxifen significant differences to the disadvantage of arm B compared to arm A (*p < *0.002) and arm C (*p = *0.006), no other significant differences in HF frequency **3:** nsd except for an increase of headache in arm B at 6 and 12 months (*p = *0.040; *p = *0.030) **4:** significant differences not in terms of physical function (*p = *0.1), but in general health favouring arm A and arm B over arm C (*p = *0.02; *p = *0.03) **AEs:** no AEs reported by the patients (pat.), increase of HF and headaches in arm B according to statistical analysis, overall incidence (any type, any grade) equally distributed between all arms
Pérol et al. (2012)	RCT	Breast-CA **arm A:** CTX, Cocculine, antiemetic therapy **arm B:** CTX + placebo, antiemetic therapy	**1:** CTX induced nausea and vomiting (CINV) in 1^st^ CTX-cycle **2:** CINV 2^nd^ and 3^rd^ CTX-cycle **3:** treatment compliance **AEs**	**1:** nsd between both arms during 1st CTX-cycle according to FLIE-questionnaire or patient diaries **2:** nsd between both arms except for significantly more vomiting episodes during 3rd cycle (assessed with patient diaries, *p = *0.030) in favour of arm A **3:** similar between arms considering patient diaries & count of remaining tablets **AEs:** no side effects related to the intervention drug
Sorrentino 2017	RCT	Breast-CA **arm A:** Arnica montana **arm B:** placebo	**1:** reduction in blood & serum volumes **2:** duration of drainage **3:** time until collected volume < 10 ml **4:** self-evaluation of pain (VAS) **5:** average time of hospitalization **6:** bruises & hematomas / breast swelling **7:** difference between volume collected on day 1 & volume on each of the following days **8:** side effects	**1:** nsd in ITT-dataset (*p = *0.60), significant reduction in blood & serum volumes in PP-dataset (*p = *0.03) **2:** neither ITT- nor PP- dataset shows significant differences (*p = *0.7287, *p = *0.8653) **3:** neither ITT- nor PP- dataset shows significant differences (*p = *0.8653, *p = *0.6138) **4:** nsd in VAS between both arms **5:** no data reported **6:** nsd regarding bruises & hematomas (*p = *0.67) or breast swelling (*p = *0.57) **7:** significant differences in favour of arm A the PP-dataset on day 2 in the univariate model (*p = *0.034) & regression modell (*p = *0.033), also on day 3 in the regression modell (*p = *0.0223) **8:** no severe side effects, not correlated with homeopathic treatment
Thompson et al. 2005	RCT	Breast-CA with HF **arm A:** indiv. Homepathy **arm B:** placebo	**1:** activity & profile score **2:** Menopausal Symptom Questionnaire **3:** Hot flash severity & frequency **4:** QoL + BreastCa-module **5:** Hospital Anxiety & Depression Scale **6:** satisfaction & perception of helpfulness **7:** Glasgow Homeopathic Hospital Outcome Scale **AEs**	**1:** nsd in activity score (*p = *0.17) or profile score (*p = *0.13) **2:** nsd in night sweat frequency, influence on sleep, days weat frequency or influence on daily functioning **3:** no data reported **4:** nsd regarding general health (*p = *0.62) or QoL (*p = *0.85) **5:** nsd regarding anxiety and depression **6:** significant differences favouring arm B (*p = *0.01) **7:** nsd **AEs:** 25% of pat. in both arms suffered AEs with only minor differences regarding aggravations, appearance of new symptoms, return of former symptoms
Frass et al. 2020a, b	RCT	NSCLC, **arm A**: indiv. homeopathy and CTX **arm B**: placebo and CTX **Arm C:** control	**1:** qol **2:** survival **3:** previous alternative treatment **4:** attitude towards Homeopahy **AEs**	**1:** significant differences favoring homeopathy (*p* ≤ 0.001) after 9 and 18 weeks for physical, role, emotional and social functioning as well as fatigue, nausea and vomiting, dyspnoea, insomnia, appetite loss as well as constipation (*p = *0.008; *p = *0.005), significant differences only after 18 (and not 9) weeks in cognitive function (*p = *0.113; *p = *0.001), pain (*p = *0.061; *p < *0.001), diarrhoea (*p = *0.590; *p = *0.017) and financial difficulties (*p = *0.134; *p = *0.021) **2:** significant differences favouring arm A in median mortality (A vs B 435 vs 257 days, *p = *0.01; A vs C 228 days, *p < *0.001; B vs C nsd) and 2-year mortality (A vs B 45.1% and 23.4%, *p = *0.020; A vs C 13.5%, *p < *0.001; B vs C nsd); significant differences regarding pat. who died within 2 years favouring arm A compared to arm C (*p = *0.020), nsd between the other arms; significant differences regarding estimated survival time (A vs B (477 vs 352 days, *p = *0.014), A vs C (274 days, *p < *0.001), B vs C nsd) **3:** nsd, mostly psychotherapy **4:** former homeopathy treatment referred by practitioners (57.1% arm A, arm B 17.6%), pat. in arm B used homeopathy significantly more often without doctors’ recommendation (*p = *0.039), expectaions of pat. in arm A was significantly lower (*p = *0.010) **AEs:** no side effects related to the intervention drug
Heudel et al. 2019	RCT	Breast-CA, **arm A:** BRN-01 (Actheane^®^) **arm P:** placebo	**1:** HF scale 1st – 2nd visit **2:** HF scale 1st – 3rd visist **3:** compliance **4:** safety & tolerance **5:** QoL **6:** satisfaction	**1:** nsd in HF scale after 4 weeks (*p = *0.756) **2:** nsd in HF scale after 8 weeks (*p = *0.775) **3:** compliance in arm A < arm P, but nsd (*p = *0.606) **4:** cases of joint pain and cholecystitis, not related to remedy **5:** nsd in QoL, no statistical analysis was made **6:** no major differences, no statistical analysis
Lotan et al. 2020	RCT	Breast-CA or risk reduction, post mastectomy & immediate breast reconstruction **arm A:** Arnica montana Bellis & perennis **arm B:** placebo	**1:** time to drain removal **2:** opioid intake **3:** QoL, quality of recovery **4:** postoperative pain (VAS) **5:** haemoglobin **6:** cortisol levels **AEs**	**1:** time to drain removal significantly shorter in arm A (11.1 ± 6.1 days in arm A; 13.5 ± 6.4 days in arm B; *p < *0.05), amputated breast weight & implant volume significantly lower in arm A (*p < *0.001) **2:** trend towards lower opioid intake in arm A, but nsd (*p < *0.057) **3:** nsd, no *p* value reported **4:** nsd in VAS, no *p* value reported **5:** nsd regarding hemoglobin, no *p* value reported **6:** nsd in cortisol levels, no *p* value reported **AES:** no side effects related to the intervention drug
Karp et al. 2016	CT	Breast-CA **Arm H:** Aromatase inhibitor + Ruta graveolens & Rhus toxicodendron **Arm C**: AI only	**1:** joint pain **2:** joint stiffness **3:** morning & daytime intensity of stiffness **4:** time to disappearance of stiffness **5:** impact of pain on sleep (quality & quantity) **6:** use of analgesics **AEs**	**1:** overall composite pain score significantly worse in arm C (*p < *0.0001), as well as frequency (*p = *0.0004), intensity (*p = *0.0004) and number of sites (*p = *0.0315) **2:** nsd regarding the overall scores for joint stiffness *p = *0.0567, joint stiffness worsened significantly more in arm C (*p = *0.0141) **3:** significantly better evolution of intensity of morning stiffness in arm H (*p = *0.0198), nsd in daytime stiffness (*p = *0.179) **4:** significantly lesser time in arm H until disappearance of morning stiffness (*p = *0.022) **5:** lower impact of JP on sleep in arm H (*p = *0.0083), no statistical analyses for patients whose pain never disturbed sleep **6:** significant differences in frequency (*p = *0.0034) and increase (*p = *0.0076) of analgesic use **AEs:** no side effects related to the intervention drug
Steinmann et al. 2012	CT	Head & neck tumours, RTX/RCTX **arm A:** Traumeel S solution **arm B:** Sage tea (Salvia officinalis)	**1:** grade of oral mucosis **2:** intraoral pain **3:** QoL **4:** xerostomia (difficulty in speech & eating) **AEs**	**1:** nsd regarding grade of oral mucosis **2:** nsd regarding intraoral pain **3:** nsd regarding QoL **4:** significant difference in week 4 favouring arm A (no p-value reported) **AEs:** no information on side effects
Clover et al. 2002	Uncon-trolled pilot out-come study	Breast CA with Hot Flushes **arm A:** HF, no Breast-CA, homeopathy **arm B:** Breast-CA, homeopathy, no TMX **arm C:** Breast-CA, homeopathy, TMX	**AEs**	**AEs:** no information on side effects
Forner-Cordero et al. 2009	Pro-spective single-armed study	Breast-CA, post unilateral breast surgery & exhibited arm-lymphedema, oral Lymphomyosot + compression hosiery, kinesiotherapy & skin care	**AEs**	**AEs:** 8 of 17 patients with treatment- emergent AE s, four pat. discontinued treatment due to AEs (1 patient each with nycturia, hypertensive crisis, right hypochondrial pain, heartburn), further AEs reported were anxiety, constipation and dry mouth
Freyer et al. 2014	Pro-spective single-armed study	Different treatment-refractory types of CA, Ruta graveolens until tumor- or clinical progression	**AEs**	**AEs:** none of the AEs considered to be directly related to remedy
Schlappack 2004	Pro-spective single-armed study	Patients with Breast-CA and RTX-induced itching, Single dose of indiv. homeopathy	**AEs**	**AEs:** no information on side effects
Thompson et al. (2002)	Pro-spective single-armed study	Different types of CA, indiv. Homeopathic treatment	**AEs**	**AEs:** reactions of homeopathic remedies in 17/57 patents: aggravation of symptoms, development of old symptoms (reported as part of the healing), transient worsening of symptoms (settled on stop of remedy intake); withdrawal of homeopathic remedy was not necessary, 1 pat. was advised to stop treatment (acute blast phase of chronic myeloid leukaemia)
Thompson and Reilly 2003	Pro-spective single-armed study	BreastCA, oestrogen withdrawal, indiv. Homeopathic treatment	**AEs**	**AEs:** 7/40 patients reported new symptoms, 10 patients reported return of old symptoms, 1 patient with a difficult aggravation of symptoms which stopped after pausing the homeopathic treatment
Gartner et al. (2012)	Retro-spective single-armed study	Different types of CA, indiv. homeopathic treatment	**AEs**	**AES:** no information on side effects

### Characteristics of included studies

Concerning all relevant studies, 2016 patients were included, of whom 1594 were analysed, due to 422 drop-outs. The age of the mostly female patients ranged from 20 to 87 years, with a mean age of 54.5 (47.9–64.9) years. Reported was the inclusion of patients with breast cancer (*N = *1448), lung cancer (*N = *213), gastrointestinal cancer (*N = *54), hematological cancer (*N = *45), head and neck tumours (*N = *40), renal cell cancer (*N = *28), sarcoma (*N = *23), pancreas cancer (*N = *9) and other types of cancer (*N = *61).

### Risk of bias in included studies

The results are presented in Table 4. Eleven of the included studies have moderate quality. Seven studies were included only for side effects and AEs due to their severe lack of methodological and reporting quality (poor quality).

**Table 4:**
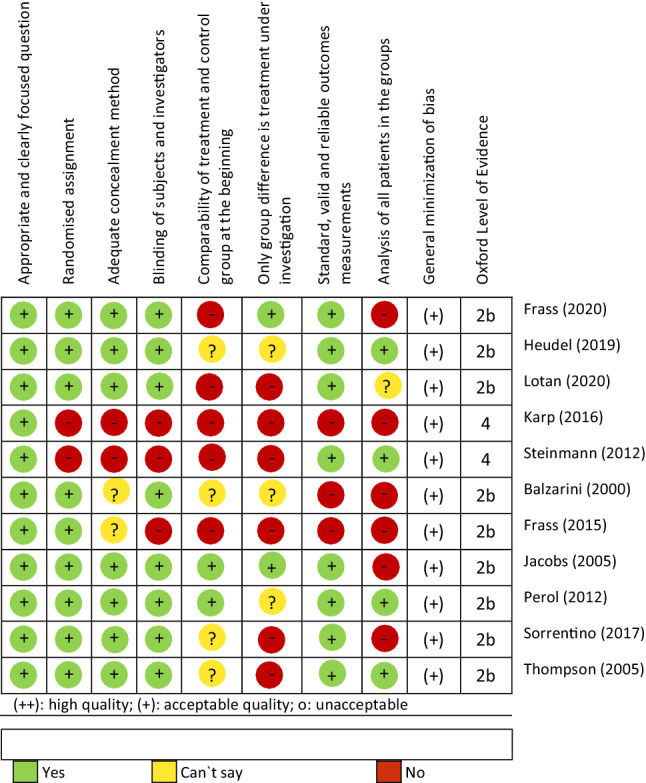
Risk of bias

### Excluded studies

A list of excluded studies after full-text screening and reasons for the exclusion can be seen in Table 5. The studies that could not be found for full-text screening (eSupplement) are listed in the appendix. One of the studies (Genre et al. 2003) was not available and our lending request remained unanswered, so we were not able to analyse the results. But while searching for the study we came across the following two reviews that had excluded the study: Mathie et al. (2013) rated the trial as a minor journal article with only an abstract available and Kassab et al. (2009) excluded the study for the following reason: “it was only available in abstract form and the results were not included in the abstract […]: the lead author was contacted but not willing to provide us with the results as the data was not published”.

**Table 5:** Excluded reviews and studies

References	Title	Reason for exclusion
T. Amidor (2020)	Ask the Expert. Colloidal Silver and Cancer	False topic
N. Aust (2016)	Prolonged lifetime by adjunct homeopathy in cancer patients-A case of immortal time bias	False topic
J. L. Bagot (2016)	Using hetero-isotherapics in cancer supportive care: the fruit of 15 years of experience	Other publication type
J. L. Bagot; N. Marechaud; N. Deana; J. Wendling (2018)	Homeopathic Treatment of Insomnia and Symptom Clusters Related to Cerebral Chemotoxicity in Oncology	Other publication type
J. L. Bagot (2020)	Homeopathy, a new tool for the prevention and treatment of chemobrain (cerebral chemotoxicity)	poor methodical quality
E. Ben-Arye; M. S. Ali-Shtayeh; M. Nejmi; E. Schiff; E. Hassan; K. Mutafoglu; F. U. Afifi; R. M. Jamous; E. Lev; M. Silbermman (2012)	Integrative oncology research in the Middle East: weaving traditional and complementary medicine in supportive care	SR, only one study about homeopathy, conducted with children
E. Bindschaedler; J. Simon (2008)	Spagyric medicine – an original way of healing with plants	False topic
A. Boyd; C. Bleakley; D. A. Hurley; C. Gill; M. Hannon‐Fletcher; P. Bell; S. McDonough (2019)	Herbal medicinal products or preparations for neuropathic pain	SR with only few studies about homeopathy, mostly herbs
A. Braillon (2020)	Expectation-based medicine in French palliative care centers: is Lyon representative?	False topic
A. Braillon; N. Ross; R. A. Fisken; E. Ernst; D. Colquhoun (2020)	Placebo therapy for cancer-related pain: an alternative to psychotherapy or health misinformation?	False topic
D. Brulé; J. Balon; Z. Linlu; D. Seely (2018)	An N-of-1 Feasibility Study of Homeopathic Treatment for Fatigue in Patients Receiving Chemotherapy	Poor methodical quality
C. P. Bryant (2014)	Observation of the End Results of Major Surgery	False topic
A. Campbell (1980)	Thuja—a drug picture based on provings	Other publication type
B. R. Cassileth (1986)	Unorthodox cancer medicine	False topic
C. W. H Chan; D. Tai; S. Kwong; K. M. Chow; D. N. S. Chan; B. M. H. Law (2020)	The effects of pharmacological and non-pharmacological interventions on symptom management and quality of life among breast cancer survivors undergoing adjuvant endocrine therapy: A systematic review	SR, majority of studies false topic or already included
A. Chatterjee; S. Bhattacharya; A. K. Chatterjee; J. Biswas; B. Mukhopadhyay (2009)	A prospective observational clinical study involving an alternative cancer treatment, psorinum therapy in treating stomach, gallbladder, pancreas, and liver cancers	False topic
A. Chatterjee; J. Biswas; A. Chatterjee; S. Bhattacharya; B. Mukhopadhyay; S. Mandal (2011)	Psorinum therapy in treating stomach, gall bladder, pancreatic, and liver cancers: a prospective clinical study	Article retracted
S. Chirumbolo; G. Bjorklund (2018)	Homeopathic Arnica from Boiron and post-operative bleeding in mastectomized women in Milan: Statistical flaws and bias to be addressed	Other publication type
K. Cseak (2019)	Cancer: My Homeopathic Method	Other publication type
A. de Nonneville; A. Goncalves (2018)	Homeopathy in cancer patients: What does the "best" evidence tell us?	Worng language (study available only in french)
S. R. Deshpande (2011)	Efficient care of challenging cases of malignancy	Other publication type
N. Djehal; J. Havas; A. Gbenou; E. Martin; C. Charles; S. Dauchy; B. Pistilli; C. Cadeau; P. Arveux; S. Everhard; J. Lemonnier; C. Coutant; P. Cottu; A. Lesur; G. Menvielle; A. Dumas; F. Andre; S. Michiels; I. Vaz-Luis; A. Di Meglio (2020)	Use of oral complementary-alternative medicine (OCAM) and fatigue among early breast cancer (BC) patients (pts)	False topic
R. Eaton (2019)	Cancer and Complementary Medicine: A Roundup	False topic
A. T. Euctr (2011)	Homeopathy in advanced malignant tumors	Other publication type
J. Filshie; C. N. J. Rubens (2006)	Complementary and alternative medicine	Other publication type
T. Fior (2019)	Summary of Kali muriaticum for the Materia Medica Pura Project	Other publication type including case studies
P. Fisher (2016)	Cancer and quality of life	False topic
P. Fisher (2018)	Individualisation and High Dilution	False topic
J. E. Flinn (1965)	Bromium in acute lymphatic leukemia	Other publication type
R. Glickman-Simon; J. Pettit (2015)	Viscum album (mistletoe) for pancreatic cancer, electromagnetic field therapy for osteoarthritis, homeopathy for multidrug-resistant tuberculosis, vitamin D for depression, acupuncture for insomnia	False topic
G. Gupta; N. Gupta; V. Singh; D. Bisht (2003)	Uterine fibroids: a clinical study with USG follow-up	False topic
H. Heine (2008)	Are homeopathic preparations of Rhus toxicodendron L. (Toxicodendron quercifolium Greene) suitable for adjuvant tumor therapy? A systematic review	Other publication type
M. Holmes (2018)	Regulatory update	Other publication type
S. Horowitz (2010)	Innovative therapies in podiatry and physical medicine	Other publication type
A. Huntley; E. Ernst (2003)	A systematic review of the safety of black cohosh	SR, false topic (not about homeopathy)
A. Karkare (2011)	Dealing with cancer	Other publication type
S. Kassab; M. Cummings; S. Berkovitz; R. van Haselen; P. Fisher (2009)	Homeopathic medicines for adverse effects of cancer treatments	High clinical heterogeneity, studies can not be summariesed, relevant sudies alsready included
M. Khoobchandani; K. K. Katti; A. R Karikachery; V. C. Thipe; D. Srisrimal; D. K. Dhurvas Mohandoss; R. D. Darshakumar; C. M. Joshi; K. V. Katti (2020)	New Approaches in Breast Cancer Therapy Through Green Nanotechnology and Nano-Ayurvedic Medicine—Pre-Clinical and Pilot Human Clinical Investigations	False topic
M. O. Kokornaczyk; S. Baumgartner (2018)	Phase Transition-Based Methods in Research on Homeopathy: A Review…HRI Malta 2017, Cutting Edge Research in Homeopathy: Presentation Abstracts	Other publication type
S. D. Klein; A. Sandig; S. Baumgartner; U. Wolf (2013)	Differences in Median Ultraviolet Light Transmissions of Serial Homeopathic Dilutions of Copper Sulfate, Hypericum perforatumy and Sulfur	Preclinical study
C. O. Lee (2004)	Integrated care. Homeopathy in cancer care: part II – continuing the practice of 'like curing like'	Other publication type
R. Leroi (1978)	Viscum album therapy of cancer	False topic
T. P. Maliekal (1997)	Antineoplastic effects of 4 homoeopathic medicines: experimental assessment	Preclinical study
F. J. Master (2006)	My experiences with cadmium metallicum, part-II	Other publication type
R. T. Mathie (2019)	Extending Homeopathy's Reach	Other publication type
R. Medhurst (2019)	Research in Homeopathy: An update	Other publication type Publication Type
F. Mehta (2011)	Iscador therapy for cancer	Other publication type
S. Milazzo; N. Russell; E. Ernst (2006	Efficacy of homeopathic therapy in cancer treatment	Studies already included
D. Milton (1998)	Alternative and complementary therapies: integration into cancer care	False topic
G. Moses (2019	What's in complementary medicines?	False topic
M. Muir (1997)	Alternative therapies for cancer-related fatigue: an introduction	Other publication type
Nct (2009	Evaluation of Questionnaires of Tumor Patients With and Without Additive Homeopathic Therapy	Other publication type
Nct (2012)	Additive Homeopathy in Cancer Patients	Other publication type
Nct (2016)	Assessment of Fatigue During Radiotherapy for Breast Cancer With and Without Homeopathy Treatment	Other publication type
Nct (2018)	Phase III Trial Evaluating Radium Bromatum Homeopathic Treatment Efficacy on Radiodermatitis Prevention and Treatment for Breast Cancer Women	Recruitment process still in progress
P. Oberai; B. Indira; R. Varanasi; P. Rath; B. Sharma; A. Soren; L. K. S. V. Bharatha; A. Sharma; P. Devi; M. Padmanabhan; V. Singh; C. Nayak (2016)	A multicentric randomized clinical trial of homoeopathic medicines in fifty millesimal potencies vis-à -vis centesimal potencies on symptomatic uterine fibroids	False topic
R. S. Pareek (1986)	Management of leukaemia and allied haematological disorders in homoeopathy	Other publication type
R. P. Patel (2008)	Cancer and the 50 millesimal potencies	Other publication type
P. Posadzki; A. Alotaibi; E. Ernst (2012)	Adverse effects of homeopathy: A systematic review of published case reports and case series	Case reports and case series only, unrealsitic results
B. Poitevin (2018)	Australian government report on the clinical effectiveness of homeopathy: Analysis and proposals	Other publication type
P. Pommier; F. Gomez; M.P. Sunyach; A. D’Hombres; C. Carrie; X. Montbarbon (2004)	Phase III Randomized Trial of Calendula Officinalis Compared With Trolamine for the Prevention of Acute Dermatitis During Irradiation for Breast Cancer	False topic
G. Rada; D. Capurro; T. Pantoja; J. Corbalan; G. Moreno; L. M. Letelier; C. Vera (2010)	Non-hormonal interventions for hot flushes in women with a history of breast cancer	False topic
J. Raphael; J. Hester; S. Ahmedzai; J. Barrie; P. Farqhuar-Smith; J. Williams; C. Urch; M. I. Bennett; K. Robb; B. Simpson; M. Pittler; B. Wider; C. Ewer-Smith; J. DeCourcy; A. Young; C. Liossi; R. McCullough; D. Rajapakse; M. Johnson; R. Duarte (2010)	Cancer pain: part 2: physical, interventional and complimentary therapies; management in the community; acute, treatment-related and complex cancer pain: a perspective from the British Pain Society endorsed by the UK Association of Palliative Medicine and the Royal College of general practitioners	Other publication type
I. L. Ray-Coquard; J. Provençal; A. C. Hardy-Bessard; T. Bachelot; D. Coeffic; J. P. Jacquin; J. P. Guastalla; C. Agostini; X. Pivot; A. Bajard; D. Pérol (2009)	Can adjuvant homeopathy improve the control of post-chemotherapy emesis in breast cancer patients? Results of a randomized placebo-controlled trial	Other publication type
J. Rosendahl; D. Jaenichen; S. Schmid; F. Farber; B. Straus (2021)	[Mental Distress and Resilience in Severe Somatic Diseases: An Analysis of Dyadic Relations]	SR with only 3 studies, only 1 about homeopathy (but no reference reported)
C. Rosser (2004)	Integrated care. Homeopathy in cancer care: part I – an introduction to 'like curing like'	Other publication type
E. Rossi; M. Picchi; C. Fonte; M. Pellegrini; E. Baldini (2016)	Integrative approach to the cancer patients with complementary medicine and diet in the Hospital of Lucca (Italy)	False topic
J. Sachdeva; J. K. Dey (2019)	Homoeopathy in Cancer Pain Palliation and End of Life with Future Perspectives	False topic
M. K. Sahani (2020)	Cracking Homoeopathic Codes In Breast Cancer	Other publication type
J. Saquib; B. A. Parker; L. Natarajan; L. Madlensky; N. Saquib; R. E. Patterson; V. A. Newman; J. P. Pierce (2012)	Prognosis following the use of complementary and alternative medicine in women diagnosed with breast cancer	False topic
S. Shah (2015)	Weeds for Cancer	Other publication type
D. Shaw; M. Frass; M. Oberbaum (2015)	A pragmatic decision to avoid blinding, placebos and disclosure of conflicts of interest: an RCT of homeopathy for cancer patients	Other publication type
E. J. Shellard (1978)	The contribution of the plant kingdom to medicine	False topic
C. Shneerson; T. Taskila; N. Gale; S. Greenfield; Y. F. Chen (2013)	The effect of complementary and alternative medicine on the quality of life of cancer survivors: A systematic review and meta-analyses	SR with only one studie about homeopathy
P. Shukla; C. Nayak; M. Q. Baig; P. Misra (2019)	A Systematic Review of Controlled Trials of Homeopathy in Adverse Effects of Radiotherapy and Chemotherapy in Cancer	SR, relevant studies already included
T. Simonart; C. Kabagabo; V. De Maertelaer (2011)	Homoeopathic remedies in dermatology: A systematic review of controlled clinical trials	SR with only one studie about homeopathy
S. Sinha; S Jain (1994)	Natural products as anticancer agents	Other publication type
S. Smith (2005)	My integrated approach to lymphoma including chemotherapy	Other publication type
J. Snyder; T. Caulfield (2019)	Patients' crowdfunding campaigns for alternative cancer treatments	False topic
S. E. Straus (2002)	Herbal medicines—What's in the bottle?	False topic
F. Talarico; C. M. Pullano; S Di Salvo; V. Falabella (2016)	Single-blind study assessing the individualized homeopathic treatment of cancer patients versus placebo	Other publication type
E. A. Thompson; R. T. Mathie; E. S. Baitson; S. J. Barron; S. R. Berkovitz; M. Brands; P. Fisher; T. M. Kirby; R. W. Leckridge; S. W. Mercer; H. J. Nielsen; D. H. K. Ratsey; D. Reilly; H. Roniger; T. E. Whitmarsh (2008)	Towards standard setting for patient-reported outcomes in the NHS homeopathic hospitals	False topic
L. Thompson (2000)	Experimental treatments? Unapproved but not always unavailable	False topic
A. Vickers (1994)	Use of complementary therapies	False topic
C. R. von Klinkenberg (2008)	The homeopathic treatment of cancer, part 2	Other publication type
P. Wood (2018)	Homeopathic Cancer Drugs: Oncology Materia Medica	Other publication type
H. V. Worthington; J. E. Clarkson; G. Bryan; S. Furness; A. M. Glenny et al. (2011)	Interventions for preventing oral mucositis for patients with cancer receiving treatment	Wrong sample (children included)
J. Wurster (2018)	Added Value of Homeopathy in Oncology	Other publication type
R. Yadav; B. Jee; K. R. S. S. Rao (2018)	How homeopathic medicine works in cancer treatment: Deep insight from clinical to experimental studies	Other publication type
B. Yanju; K. Xiangying; Y. Liping; L. Rui; S. Zhan; L. Weidong: H. Baojin; H. Wei (2014)	Complementary and Alternative Medicine for Cancer Pain: An Overview of Systematic Reviews	Other publication type
C. Yde; P. Viksveen; J. Duckworth (2019)	Reasons for Use of and Experiences with Homeopathic Treatment as an Adjunct to Usual Cancer Care: Results of a Small Qualitative Study	Poor methodical quality
J. M. Young; D. Jusufbegovic (2019)	Extramedullary Retinal Involvement in Chronic Myeloid Leukemia	False topic

### Efficacy of homeopathic therapy

#### Influence on toxicity of cancer treatment: skin reaction

Balzarini et al. (2000) analysed the effects of Belladonna 7CH globules (two times a day) and X-ray globules (once a day) associated in the treatment of acute radiodermatitis compared to a placebo in 61 randomized breast cancer patients. Over 30 days after radiotherapy the physician assessed skin color, temperature to the touch, edema and hyperpigmentation at eight defined times (*t*1–*t*8). There were no differences in skin color (all *p*’s > 0.050) and hyperpigmentation (all *p*’s ≥ 0.050) but the study found significant differences in temperature for *t*3, *t*4, *t*6 and *t*7 (*p = *0.008; *p = *0.016; *p = *0.023; *p = *0.011) in favour of the homeopathy group. They also found a difference for oedema on at *t*5 and *t*6 in favour of the placebo group (*p = *0.025; *p = *0.025).

#### Influence on toxicity of cancer treatment: nausea and vomiting

Pérol et al. (2012) included 403 breast cancer patients in a RCT to investigate chemotherapy-induced nausea and vomiting. Patients in the intervention group took the complex homeopathic remedy “Cocculine”, while the control group was given a placebo in addition to the standard antiemetic therapy during six chemotherapy cycles. Instruments to assess nausea and emesis were the Functional Living Index for Emesis questionnaire, patient diaries and the Common Terminology Criteria for Adverse Events Scale. There was no significant difference between the arms during first, second or third chemotherapy cycle (all *p*’s > 0.050), except for significantly more vomiting episodes during third cycle (assessed with patient diaries, *p = *0.030) in favour of the homeopathy arm.

#### Influence on toxicity of cancer treatment: joint pain (JP) and joint stiffness (JS)

In an open, not randomized CT by Karp et al. (2016) 27 breast cancer patients were included, taking only aromatase inhibitors in the control group or additionally Ruta graveolens 5CH and Rhus toxicodendron 9CH (twice a day for 3 months) in the homeopathic group. The overall scores showed a significant advantage in the homeopathic arm for JP (*p = *0.000) but not for JS (*p = *0.057). More results of significance, all in favour of the homeopathy arm, were frequency, intensity and number of sites regarding JP (*p = *0.000; *p = *0.000; *p = *0.032), morning (not daytime) intensity, worsening of JS and time to disappearance of morning stiffness and (*p = *0.020; *p = *0.179; *p = *0.014; *p = *0.022) as well as frequency and increase of analgesic use concerning JP (*p = *0.003; *p = *0.008). At inclusion, 65% and 80% of patients in the homeopathic and control arm complained of JP, whereas 76.9% and 62.5% had taken analgesics in the week before inclusion.

#### Influence on toxicity of cancer treatment: oral mucositis

Another non-blinded and not randomized CT by Steinmann et al. (2012) analysed the grade of oral mucositis in 20 patients with head and neck tumours receiving radiotherapy or radio-chemotherapy. Patients in the homeopathic arm carried out mouth rinses with a Traumeel S solution, the control group with sage tea (Salvia officinalis) for 6–7 weeks. The authors found no significant differences in the grade of oral mucositis between both groups (no *p* values given) and reported a consistent worsening of intraoral pain during the study period, except for one single time in week 5 in the homeopathic arm. At the end of the study, 6 and 3 out of 10 patients took systemic analgesics in the homeopathic and placebo arm, while 5 and 1 out of 10 patients used local analgesics, but no statistical analysis was made. Regarding xerostomia (difficulty in speech and eating), they reported a significant difference in preservation of taste favouring Traumeel in week 4, but presented no *p* value.

#### Influence on toxicity of cancer treatment: influence of JP on sleep

Twenty-seven breast cancer patients were assessed regarding the impact of JP on quality and quantity of sleep in an open, not randomized CT by Karp et al. (2016). While patients in the control group were taking aromatase inhibitors only, patients in the homeopathic group received additionally Ruta graveolens 5CH and Rhus toxicodendron 9CH (twice a day for 3 months).

The impact of JP on sleep after 3 months showed a significant difference in favour of the homeopathy arm (*p = *0.008). No statistical analyses were done for the results of patients who stated that pain never disturbed their sleep.

#### Time to drain removal after mastectomy

A RCT by Luca Sorrentino et al. (2017) observed 53 breast cancer patients (intention to treat (ITT)-sample; in the per protocol (PP)-sample 43 patients) who were either taking Arnica montana 1000 K or a placebo (3 times a day) from one day before until 4 days after surgery. The results of reduction in drained blood and serum volumes were analysed with three different models.

Regarding the changes in volume collected from day one, analysed with the analysis of variance (ANOVA), neither the PP- nor the ITT- sample showed significant overall differences (*p = *0.772; *p = *0.122). When analysed with the regression model including treatment and collected volume on the day of intervention, the differences between the groups in the PP-sample were significant on days 2 and 3 to the advantage of homeopathy (*p = *0.033; *p = *0.022). The estimates of the mean difference in total volume analysed with regression models showed significant differences only in the PP-sample for the model including treatment, collected volume on the day of surgery and patient weight (*p = *0.030). The differences in the ITT- sample were not significant (*p = *0.600).

Regarding self-evaluation of pain**,** bruises and haematomas or breast swelling after surgery both arms showed no significant differences (*p* > 0.050; *p = *0.670; *p = *0.570).

Fifty-five patients with breast cancer or risk patients wishing for risk reduction by undergoing mastectomy and immediate breast reconstruction were assessed in a RCT (Lotan et al. 2020). Patients were either taking three globes of Arnica montana Bellis C30 & perennis C30 each or a placebo until drain removal. Concerning this matter, a significant difference favouring homeopathy was found (11.1 ± 6.1 days in study group, 13.5 ± 6.4 days in placebo group, *p < *0.050), but because the amputated breast weight and implant volume may affect drainage and differed significantly between both groups (*p < *0.001), this result cannot be fully attributed as intervention effect. Concerning postoperative pain, haemoglobin, opioid intake and cortisol levels, no significant differences were found.

#### Survival

Frass et al. (2020a, b) observed 150 randomized patients with advanced non-small cell lung cancer until death or in case of survival for a maximum of 24 months. Fifty-two patients gave no consent to randomization and were, therefore, used as a control group for this endpoint only (arm C), while the other groups received chemotherapy and either individualized homeopathic medicine (daily on a 3-week interval, arm A) or a placebo (arm B). Over the observed 2 years, median- and 2-year mortality differed significantly between arm A and B (435 and 257 days, *p = *0.010; 45.1% and 23.4%, *p = *0.020), arms A and C (228 days, *p < *0.001; 13.5%, *p < *0.001) but not between arms B and C (*p = *0.258; *p = *0.154). Further significant differences were found for the estimated survival time between arms A and B (477 and 352 days, *p = *0.014), arms A and C (477 and 274 days, *p < *0.001) but not arm B vs arm C (*p = *0.145), as well as for patients who died within the 2 years (A vs C, *p = *0.020; not A vs B *p = *0.172 and B vs C *p = *0.747).

#### Hot flashes (HF) and other menopausal symptoms

To explore the effect of homeopathy on HF, Jacobs et al. (2005) conducted a randomized study with 66 breast cancer patients receiving either a placebo combination medicine and a homeopathic single remedy (arm A), a homeopathic combination medicine (Hyland’s Menopause) and a placebo single remedy (arm B) or 2 placebos (single and combination remedy, arm C). The overall results regarding severity and frequency of HF and typical menopausal symptoms (via Kupperman Menopausal Index) did not differ significantly, except for an increase of headache in arm B at 6 and 12 months (*p = *0.040; *p = *0.030). A subgroup analysis including only patients without tamoxifen regimen showed significant differences, arm B, in HF severity score (frequency times severity: B vs C *p = *0.010, A vs B *p < *0.001) and in the total number of HF (B vs C *p = *0.006, A vs B *p = *0.002). Furthermore, patients in arm A had a lower severity score and fewer HF in total.

Assessing 53 breast cancer patients, a RCT by Thompson et al. (2005) did not find any significant differences in activity- and profile-scores (all *p*’s > 0.05) between the intervention group receiving individual homeopathic treatment for 16 weeks and the placebo group. No significant differences were found in menopausal symptoms (conducted through a questionnaire) as well, assessing night sweats frequency and influence on sleep (*p = *0.750; *p = *0.870) and day sweats frequency and disturbance of everyday functioning (*p = *0.300; 0.220). Only the differences in terms of satisfaction were significant, but in favour of the placebo group (*p = *0.010). On HF -severity and -frequency no data were reported.

In another study, 138 randomised patients took the homeopathic remedy BRN-01 (Actheane^®^) or a placebo twice a day for at least 8 weeks in addition to their adjuvant endocrine therapy (aromatase inhibitor or tamoxifen with/without ovarian suppression). There were no significant differences in the HF-score after 4 or 8 weeks (*p = *0.756; *p = *0.775), compliance (*p = *0.606) or satisfaction (Heudel et al. 2019).

#### Quality of life (QoL), quality of recovery (QoR), global health and subjective well-being

The influence of homeopathy on improving the global health status or subjective wellbeing was assessed in an RCT by Frass et al. (2015). For an unstated duration, 373 unblinded patients with different kinds and stages of carcinoma received either chemotherapy or radiotherapy only or an additional individual homeopathic treatment. After 4 months, the arms showed significant differences in global health (via EORTC QLQ-C30, *p = *0.005) and subjective wellbeing (via visual analogue scale (VAS), *p < *0.001) favouring homeopathy.

Assessing 150 patients with advanced non-small cell lung cancer (NSCLC) receiving chemotherapy and an individualized homeopathic treatment or a placebo, the authors found comparable results in their RCT in 2020 after 9 and 18 weeks in global health status/QoL (*p < *0.001) and subjective well-being (via SF-36, *p < *0.001) (Frass et al. 2020a, b). In both trials, most of the assessed function- and symptom- scales showed significant differences favouring homeopathy after 4 months (Frass et al. 2015): *p < *0.001 for physical, cognitive, social and emotional functioning as well as fatigue and pain; role functioning *p = *0.040, dyspnoea *p = *0.002, insomnia *p = *0.029, appetite loss *p = *0.007) and after 9 and 18 weeks (Frass et al. 2020a, b: *p* ≤ 0.001 for physical, role, emotional and social functioning as well as fatigue, nausea and vomiting, dyspnoea, insomnia, appetite loss as well as constipation (*p = *0.008; *p = *0.005). Significant differences only after 18 (and not 9) weeks were found in cognitive function (*p = *0.113; *p = *0.001), pain (*p = *0.061; *p < *0.001), diarrhoea (*p = *0.590; *p = *0.017) and financial difficulties (*p = *0.134; *p = *0.021). The results for vomiting and nausea, constipation and diarrhoea in the study by (Frass et al. 2015) did not reach significance.

Patients with former homeopathic experience were surveyed regarding their attitude concerning homeopathy by Frass et al. 2020a, b) in their study on patients with NSCLC. The majority of patients in the study arm receiving homeopathy had been referred to the former homeopathic treatment by doctors (57.1%, arm B 17.6%) and their expectations regarding a homeopathic effect were significantly lower (*p = *0.010) than the expectations of patients in the placebo arm, who had significantly more often used homeopathy without a doctor’s recommendation (*p = *0.039).

In a RCT by Jacobs et al. (2005) 66 breast cancer patients were analysed and received either a placebo combination medicine plus a homeopathic single remedy (arm A), Hyland’s Menopause (a homeopathic combination medicine) plus a placebo single remedy (arm B) or 2 placebo medications (arm C). After 1 year the study found significant results in QoL not in terms of physical function but in general health (via SF-36) favouring both homeopathic arms A and B over placebo (*p = *0.020; *p = *0.030).

Further studies observing QoL did not find significant differences: neither in a controlled trial with 20 non-blinded and non-randomized patients with head and neck tumours (Steinmann et al. 2012), no *p* values reported) receiving Traumeel S or sage tea for mouth rinses against radiotherapy- or radiochemotherapy- induced oral mucositis, nor in a RCT with 138 patients who took, additionally to their adjuvant endocrine therapy, the homeopathic remedy BRN-01 (Actheane^®^) or a placebo (Heudel et al. 2019). In the latter study no statistical analysis was made between the groups and the result presentation was incomprehensible.

Two RCTs (Lotan et al. 2020; Thompson et al. 2005) found no significant differences in general health, QoL or QoR comparing the effects of Arnica montana and an indiviualized homeopathic remedy to a placebo (no *p* value reported; *p = *0.850).

#### Anxiety and depression

This endpoint was assessed by Thompson et al. (2005), who found no significant differences for anxiety and depression between the homeopathic and placebo arm in 53 randomized breast-cancer patients.

#### Safety, tolerance and side effects

Two studies analysed safety and side effects as one of their secondary endpoints.

The reported adverse events (AEs) in the RCT by Luca Sorrentinoet al. (2017) by five patients taking Arnica montana were not correlated with the homeopathic treatment. None of the AEs stated in another RCT were related to the study treatment with BRN-01 (Actheane^®^) or the placebo, as well (Heudel et al. 2019).

Six studies reported no side effects related to the intervention drug (Frass et al. 2015; Frass et al. 2020a, b; Freyer et al. 2014; Karp et al. 2016; Lotan et al. 2020; Pérol et al. 2012). Further four studies (Clover and Ratsey 2002; Gaertner et al. 2014; Schlappack 2004; Steinmann et al. 2012) gave no information on side effects of the study remedies. Because the studies assessed the homeopathic treatment during cancer care, it was often impossible to define the exact cause of the reported AEs. Balzarini et al. (2000) reported one drop-out due to homeopathic exacerbation (Belladonna 7cH globules, two times a day and X-ray globules once a day) and four drop-outs due to the AE’s of radiation.

In another study (Jacobs et al. 2005) there were no AEs reported by the breast cancer patients receiving a placebo combination medicine and a verum single remedy in arm A, a verum combination medicine (Hyland’s menopause) and a placebo single remedy in arm B or 2 placebo medications in arm C. But statistical analysis showed an increase of HF and headaches in arm B although the overall incidence (any type, any grade) was equally distributed between all groups.

Thompson et al. (2005) reported that about 25% of patients in both groups (receiving an individualized homeopathic remedy or a placebo) suffered side effects with only minor differences in terms of aggravations, appearance of new symptoms or return of former symptoms. Details about severity, kind of AE and whether they relate to the remedies were not given.

Further seven studies were included for side effects (Clover and Ratsey 2002; Forner-Cordero et al. 2009; Freyer et al. 2014; Gaertner et al. 2014; Schlappack 2004; Thompson and Reilly 2002; 2003). Of these, two studies reported no information about AEs and were, therefore, mentioned in the listing above (Gaertner et al. 2014; Schlappack 2004).

A study by Forner-Cordero et al. (2009) analysed 17 breast cancer patients after unilateral breast surgery with exhibited arm- lymphedema, who were treated with oral Lymphomyosot (15 drops or 3 tablets) for three times a day over the study period, in combination with compression hosiery, daily kinesiotherapy and skin care. Eight patients experienced treatment-emergent AE ‘s and four patients had to discontinue their treatment due to AEs (one patient each with nycturia, hypertensive crisis, right hypochondrial pain, heartburn, no further information given). Further AEs reported were anxiety, constipation and dry mouth.

Another study by Thompson and Reilly (2002) reported reactions of homeopathic remedies that were given according to individual assessment in 17 of 57 patients with different cancer types receiving conventional cancer treatments. Reactions included aggravation of symptoms, development of old symptoms from years ago (reported as part of the healing) and transient worsening of symptoms (which settled on stopping the remedy). None of the AEs necessitated withdrawal of homeopathic medicines, but one patient was advised to stop the treatment because of an acute blast phase of chronic myeloid leukaemia.

In 2003 the authors assessed individualised homeopathic medicine in breast cancer patients under conventional cancer therapy and reported new symptoms in 7 of 40 patients, return of old symptoms in 10 patients and 1 patient suffering a difficult aggravation of symptoms which stopped with pausing the homeopathic treatment (no further information given) (Thompson and Reilly 2003).

## Discussion

Before summing up the main results it should be noted that due to the variety of remedies, potencies and indications used in the included studies, finding evidence of the effectiveness of homeopathic treatment in cancer patients is problematic. Patients receiving individualized and changing homeopathic treatment even within a single study generate difficulties in deriving results for certain symptoms. As heterogeneous as the homeopathic agents were the types of cancer and, consequently, the conventional anti-cancer therapies, leading to many different observed endpoints.

All of the included studies showed strong methodical deficits in study design and reporting of the data such as incomplete description of sample, patient characteristics, drop-out, dose, duration of intervention or statistical data.

Regarding the influence of homeopathy on toxicity of cancer treatment, one study analysed skin reactions of irradiation (Balzarini et al. 2000) and obtained conflicting results both to the advantage and disadvantage of homeopathy which may have been biased by the small sample size. The authors reported a trend of less dermatitis and for one assessment (t5) interpreted a *p = *0.05 wrongly as significant in favour of the homeopathy group. It remains unclear why the authors used invalid scores instead of internationally accepted and valid scores (Radiation Therapy Oncology Group -score for example).

One study by Karp et al. (2016) addressed the homeopathic influence on JP and JS caused by aromatase inhibitors. Patients who received homeopathic treatment were reported to have a significantly greater improvement in all results concerning JP and analgesic use. Contrary to this, only a few measurements were significant (mean time to disappearance of JS, morning intensity and worsening of JS). Strangely, more patients in the control group stated JP at inclusion, but took less analgesics than patients in the homeopathic group. The analgesic consumption, however, was not properly described at materials and methods. Moreover, the study shows severe methodological weaknesses: both arms were unblinded, not randomized and important inclusion criteria, such as cancer stage, are not mentioned. The authors report only few *p* values that mostly refer to the composite scores for joint pain and joint stiffness, leading to highly significant *p* values. But these scores are not valid and seem questionable. Moreover, the generated percentages are based on different baseline values. For their calculations, the authors seem to use either two different numbers of patients at inclusion for each study arm or the number of patients after 3 months. It remains incomprehensible and unreported which dataset is used for which endpoint and some calculated results stay questionable. Also, some numbers reported in the text differ from those in the tables. Furthermore, the comparability of both groups is questionable: each group was treated at a different hospital and patients showed severe differences at inclusion already. Besides, the drop-out was high and differed in both arms (homeopathy arm 45%, control arm 20%).

Chemotherapy-induced nausea and vomiting (Pérol et al. 2012) as well as oral mucositis during radiotherapy or radio-chemotherapy (Steinmann et al. 2012) were studied in one trial only, and both were unable to find a homeopathy effect. Vomiting episodes that Perol et al. reported significantly more often in the placebo group during the 3rd chemotherapy cycle, were not obtained over the 4–6th cycle and had no impact on the Functional Living Index. Although Steinmann et al. reported a significant advantage for the homeopathic group regarding preservation of taste in week 4, the authors provided no data on significance for this statement that was based on diaries of the 20 patients. Furthermore, the use of systemic and local analgesics was higher in the homeopathy group compared to the control group. Whether this is the result of harm caused by the homeopathic remedy or other reasons remains unclear.

Only one study assessed the influence on JP on quality and quantity of sleep (Karp et al. 2016). The patients in this controlled trial received either aromatase inhibitors only or additionally Ruta graveolens 5CH and Rhus toxicodendron 9CH. To the benefit of homeopathy, the study showed a significantly worsened impact of pain on sleep concerning JP in the placebo group after 3 months, while the homeopathy group remained unchanged. Regarding the results of patients whose sleep was never disturbed by pain, no statistical analyses were done. However, the authors use different baseline values for their calculations and it remains incomprehensible and unreported which dataset is used for which result. Also, the patients in this study were neither blinded nor randomized and important inclusion criteria, such as cancer stage, was not reported. Furthermore, the patients in the study arms showed strong differences right from the start and were treated at two different hospitals, which limits the comparability. Additionally, the drop-out was high and uneven (homeopathy arm 45%, control arm 20%).

Inconsistent findings were obtained in two blinded and placebo-controlled studies assessing the effects of homeopathic interventions on time to drain removal in breast cancer patients after mastectomy (Lotan et al. 2020; Luca Sorrentino et al. 2017). Luca Sorrentino et al. (2017) reported significant differences favouring homeopathy in two different regression models of the per-protocol-analysis only: in total volume (including treatment, collected volume on day of surgery, patient weight) and in changes in volume collected from day 1 to each following day in two time points (including treatment, collected volume on day of surgery). Yet, neither the overall results in the ANOVA—nor the regression- model of the ITT-sample did reach significance. The study lacks reporting quality: only few baseline characteristics are described, details on cancer stage are missing and the reporting of results for the endpoints is incomplete. The comparability of both study arms is questionable due to missing detail about whether mastectomy was performed with or without reconstruction, which most likely affects the amount of volume. Most importantly, the authors do not report whether both arms of the PP-analysis are comparable to the baseline data or not. That is why the results of the PP-dataset are not usable. Furthermore, the high and uneven drop-out (homeopathic arm 12%, placebo arm 26%) and the small sample size (53 patients) may have biased the PP-dataset and limits the generalizability of the results even more.

Lotan et al. (2020) reported significant advantages for the homeopathic group, but included patients for therapeutic as well as prophylactic mastectomy which may have gone along with different radicality of the operation in both arms. Also, the volume of the operated breast and the implant were different in both arms. This, and a different radicalism of the operation, most likely affected the drained volume and postoperative complications and biased the outcome. Additionally, the durations until drain removal in the results are in contrast with the range of drain times stated in the limitations (3–32 days). Further severe inconsistencies are the changed trial protocols during the study, as well as the uneven compliance and drop-out of patients. They were kept in the statistical analysis as partially treated, but no data were reported. Besides, only few patient characteristics are stated. Last but not least, the authors either report wrong numbers or transposed them. Further limitations of this study were discussed by the authors. Hence, these trials cannot serve as evidence for the effectiveness of homeopathic treatment in breast cancer patients.

Frass et al. (2020a, b) conducted the only study observing the use of a homeopathic treatment on survival among patients with advanced non-small-cell lung cancer. Significant differences in median-, 2-year- mortality and estimated survival time were found favouring a homeopathic over a placebo and a not randomized control group. But as discussed by the authors the comparability between the arms is restricted as there were significantly more patients with N (Nodus) stages 0–1 in the placebo arm, and more patients with N stage 3 in the homeopathic arm (*p = *0.010). Furthermore, there are serious concerns with respect to the reporting of this study: the authors gave contradictory statements in the text and study protocol on whether the control group, that refused randomization, was given verum or not. Additionally, the high and uneven drop-out (homeopathy 9.8%, placebo 29.8%, no data for control group) might be the result of some selection bias. A serious concern also is the unusually high number of deaths in the first weeks in the placebo group, for which there is no explanation. The fact which is most serious concerning the scientific conduct of the study is the fact that the trial protocol has been changed for several times. This is well documented as the study was registered in clinicaltrials.gov (“https://clinicaltrials.gov/ct2/show/ NCT01509612?term = 33010094 + %5BPUBMED-IDS%5D&draw = 2&rank = 1”). Instead of 3 pre-planned only data on patients with one cancer type was reported, instead of 600 participants as stated in the registration only 150 were included in the final manuscript of the study while the number of exclusion criteria was raised from 1 to 20. The date of a document with modifications (January 2011), is set a year before the study was first registered in January 2012, but already contains changed parameters similar to those in the published paper (but lists 300 patients to include). Moreover, the planned follow-up was reduced from 104 to 18 weeks.

Three studies assessed the influence of homeopathic interventions on HF and menopausal symptoms. Two of the studies, that were placebo-controlled and double-blinded, demonstrated no significant effect on HF or menopausal symptoms (Heudel et al. 2019; Thompson et al. 2005). On the contrary, according to Thompson et al. (2005), patients receiving homeopathy were, to a significant degree, even more unsatisfied with the treatment than the placebo group.

Contrary to this, in a subgroup without tamoxifen regimen, a three-armed, placebo-controlled, blinded study (Jacobs et al. 2005) showed a significant increase in the total number of HF in arm B (homeopathic combination medicine (Hyland’s Menopause)) compared to arm C (2 placebos) and compared to arm A (placebo plus an individualized homeopathic single remedy). Whether or not that was the result of a harmful impact of the homeopathic combination remedy is not discussed by the authors. The study also showed a lower severity score and fewer HF in total in patients in arm A. The *p* values for mean difference of HF severity score also showed significance to the disadvantage of arm B, but looking at the confidence intervals the calculated significance is highly uncertain. The comparison of the single homeopathic remedy and a placebo did not reach statistical significance. While the patients in arm B showed a higher number of HF, had a worse HF severity score and an increase of headache, they showed, just as the single homeopathic remedy, a significantly improved general health score (via SF-36) compared to the placebo group after 1 year. Strangely, the non-responding placebo group did not receive significantly more changes of prescription. These inconsistencies might be the result of numerous methodological weaknesses of the study: most importantly, the high and uneven number of patients that had dropped out at 12 months (single remedy 36.7%, combination remedy 23.1%, placebo 40.7%), although all of the randomized patients were analysed. Methodologically questionable is the inclusion of patients with only 3 HF per day, which leaves only a low potential for improvement. It remains unreported whether patients in arm A had taken the remedy before the first telephone interview (after 1 month) because it was mostly given monthly or every 2 months. Furthermore, the patients were analysed in small subgroups with only ten patients in some groups. Baseline data for the endpoints are missing, and many results were (most likely due to missing significance) not reported at all. Therefore, the statements in this study should be viewed with caution.

Six studies investigated the effect of homeopathic interventions on QoL and QoR. Two trials reported a positive influence on global health status and subjective wellbeing (Frass et al. 2015; Frass et al. 2020a, b). Significant differences were found for the majority of the assessed function- and symptom-scales after 4 months or 9 and 18 weeks, which were valued subjectively by the patients themselves. Contrary to this, Jacobs et al. (2005) reported an effect of homeopathy regarding QoL only in general health, but not in physical function. Four studies (Heudel et al. 2019; Lotan et al. 2020; Steinmann et al. 2012; Thompson et al. 2005) did not show a significant effect on QoL or QoR.

Again, the seemingly positive studies have numerous methodological weaknesses. The patients in the trial by Frass et al. (2015) were unblinded and not compared to a placebo- or active control group. Moreover, the results were reported for patients without chemotherapy and metastases, while the authors state that 24.4% of the patients had metastases and 49.1% received chemotherapy. Furthermore, the VAS used in the study is not a valid score. The authors used multiple imputation models without reporting the quantity of the calculated missings. Taking a closer look, 37 out of 410 randomized patients dropped out, leaving 373 patients to receive study treatment. Only 335 completed the questionnaires at the first and second visit and only 282 patients completed the third visit, while 373 patients were analysed. Thus, about 10% of the data for the second visit was imputed, about 24% for the third. Considering the high dropout (homeopathic arm 34.8%, control arm 27.5%) and the different attention between groups, the multiple imputation techniques that were used might have led to incorrect results: patients in the homeopathic arm (who might expect an improvement in well-being due to the remedy or talks to a homeopath) are more likely to drop out because of disappointment than patients in the control arm (mostly taking part to support science). Perhaps because these results were more pleasant for the authors, they compared only visit one and three and did not report the results of the second visit.

The second study by the author Frass et al. 2020a, b) has been discussed above— due to the serious concerns on that study, also the data on QoL do not provide sound evidence. The follow-up for QoL changed from 2 years to 18 months. Likewise, as mentioned already, the drop-out in the study by Jacobs et al. (2005) was high and uneven (single remedy 36.7%, combination remedy 23.1%, placebo 40.7%) and might have, together with the small sample size of the subgroups (ten patients only in some groups) biased the results.

No effect of homeopathy was found regarding anxiety and depression by Thompson et al. (2005), the sole study in this review assessing that endpoint and lacking report quality.

All in all, our systematic review does not provide any evidence on the effectiveness of homeopathy in cancer care that is higher than a placebo effect.

As in higher dilutions there is no substance left any more, this result is in accordance with scientific knowledge. Accordingly, we doubt that any further well-conducted studies will come to another result. Some physicians may be inclined to use homeopathy as a placebo due to its high acceptance and reputation in the society and for patients. This makes it much easier to use the placebo effect than prescribing an unknown receipt. Moreover, homeopathy seemingly has no strong side-effects. Yet, lower dilutions may contain an amount of the substance that may lead to allergies or other side effects. Mostly, these effects will be small. Yet, this seeming advantage is no argument to justify the use of homeopathy as a placebo. Patients having a positive experience with homeopathy and other CAM tend to use these ineffective methods also in case of serious diseases (Huebner et al. 2014). Also, any delay in symptom management during cancer treatments in favour of a homeopathic treatment goes along with a deterioration of the patient’s supportive management.

Important to know, homeopaths have their own interpretation of symptoms going on or even increasing while the patient is taking homeopathy: initial worsening allegedly is a proof of the correct choice of the homeopathic remedy. For cancer patients, this idea is highly dangerous as it may lead to a further delay of treatment. Such worsening has been reported and misinterpreted in several studies in our review (Balzarini et al. 2000; Jacobs et al. 2005; Thompson et al. 2005; Thompson and Reilly 2002; 2003).

### Limitations of this work

This systematic review exhibits some limitations that must be mentioned. As listed in the exclusion criteria in Table 1, studies concerning children or teenagers were excluded and only trials with adult patients were analysed in this SR. Excluded were also other publication types than primary investigations or reports; preclinical studies, case reports or gray literature such as ongoing studies, unpublished literature, conference articles, abstracts, comments or letters. Besides, we included only studies in English or German language, leaving possible studies in other languages unconsidered. Furthermore, we could not conduct a meta-analysis. The essential reason for this is the large heterogeneity of the included studies, which was already described in the beginning of the discussion. We had to compare trials with differing design, endpoints, homeopathic intervention, type of cancer, cancer stage or cancer care to gain a comprehensive overlook. Besides, most of the subgroups were small and the majority of studies had a high risk of bias. The points mentioned would have limited the quality of a meta-analysis severely so we decided to summarize the included studies as a systematic review.

## Conclusions

All in all, the results for the effectiveness of homeopathy in cancer patients are heterogeneous, mostly not significant and fail to show an advantage of homeopathy over other active or passive comparison groups. No evidence can be provided that homeopathy exceeds the placebo effect. Furthermore, the majority of the included studies shows numerous and severe methodological weaknesses leading to a high level of bias and are consequently hardly reliable. Therefore, based on the findings of this SR, no evidence for positive effectiveness of homeopathy can be verified.

## Supplementary Information

Below is the link to the electronic supplementary material.

Supplementary file1 (DOCX 19 kb)

## Data Availability

Not applicable.
