# Supplementary material for: Homeopathy effects in patients during oncological treatment: a systematic review
Source: J Cancer Res Clin Oncol. 2022 Jun 22;149(5):1785–810. doi: 10.1007/s00432-022-04054-6 (PMC10097733; doi:10.1007/s00432-022-04054-6)
Supplement: Supplementary file 1 — Supplementary file1 (DOCX 19 kb) [file 432_2022_4054_MOESM1_ESM.docx]

**Table 6: Full-text articles not available**

| **Authors** | **Year** | **Title** |
| --- | --- | --- |
| L. Alexander | 2003 | Working with cancer patients: nutritional and complementary approaches |
| Anonymous | 1991 | Discussion on natural healing methods |
| P. Aphale | 2017 | Fight against Cancer -The Lost Hope!!! |
| M. Aspinwall | 2009 | Comfort remedies: relief for chemo, radiation, and surgery side effects |
| M. Baum | 1991 | Bridging the gulf |
| M. Bhartiya | 2015 | An Unprecedented Gesture |
| P. Bhatnagar | 2003 | Cancer |
| A. Braillon; F. Taiebi | 2018 | Prescription in oncology: Homeopathy or psychotherapies and nicotine substitutes? |
| A. K. Chimthanawala | 2017 | Managing Cancers -- Our Experiences |
| A. Clover; S. Kassab | 1998 | Complementary medicine for patients with cancer |
| K. H. Gebhardt | 1982 | Tumor treatment in general practice |
| D. Genre; C. Tarpin; A. C. Braud; J. Camerlo; C. Protiere; F. Eisinger | 2003 | Randomized, double-blind study comparing homeopathy (cocculine) to placebo in prevention of nausea/vomiting among patients receiving adjuvant chemotherapy for breast cancer |
| G. Gupta; R. Singh | 2016 | Evidence Based Study on the Effectiveness of Homoeopathic Medicines in Large Fibroids |
| K. Hamilton | 2001 | Complementary and alternative medicine in cancer research |
| G. R. Henshaw | 1963 | The unforeseen encountered in the practice of medicine |
| L. M. Khan | 2002 | Cancer: it's homoeopathic concept and treatment |
| U. R. Kleeebrg | 1986 | [Cancer drugs of questionable effectiveness] |
| R. Ludtke; J. Jacobs; E. A. Thompson | 2005 | Classical homeopathy - Much dispute about its benefits in breast cancer survivors |
| S. Maclennan | 2020 | Monty the magnificent |
| F. J. Master | 2008 | Homeopathy in cancer |
| F. J. Master | 2011 | Homeopathy in cancer |
| F. J. Master | 2013 | Radiation therapy and homeopathy |
| T. H. Neha | 1999 | Liver tumours-comparative profile of Chelidonium majus, Hydrastis, and Myrica cerifera |
| R. R. Patel | 2017 | Breast Cancer. What to do? Views of Allopathy and Homoeopathy |
| B. E. Pengelly | 2006 | Dialysis cruising |
| J. Rozencwajg | 2018 | Homeopathic Cancer drugs. Oncology Materia Medica |
| B. Rudge; M. Pacifico; D. Jallali; A. Grobbelaar | 2005 | Minerva |
| W. Sampson | 1996 | Does homeopathy work? |
| P. Sankaran | 2013 | The homoeopathic approach to cancer |
| E. Schluren | 1982 | Contribution of homeopathy to the treatment of cancer patients |
| R. Schmidt | 1963 | HOMEOPATHIC FAILURES? |
| R. Seitschek | 1981 | Possibilities and limitations of homeopathic therapy |
| R. Seitschek | 1981 | Demonstration of efficacy of homeoisotherapy in cancer |
| P. Shukla; R. Misra | 2011 | Breast lumps and homeopathic management |
| A. Singh | 2008 | Cancer. Malignant tumors |
| A. Singh | 2011 | Cancer and precancerous state |
